# A growing battlefield in the war against biofilm-induced antimicrobial resistance: insights from reviews on antibiotic resistance

**DOI:** 10.3389/fcimb.2023.1327069

**Published:** 2023-12-19

**Authors:** Liu Pai, Sandip Patil, Sixi Liu, Feiqiu Wen

**Affiliations:** ^1^ Department of Hematology and Oncology, Shenzhen Children’s Hospital, Shenzhen, China; ^2^ Pediatric Research Institute, Shenzhen Children’s Hospital, Shenzhen, China

**Keywords:** biofilms, diagnosis, antimicrobial resistance, infections, treatment

## Abstract

Biofilms are a common survival strategy employed by bacteria in healthcare settings, which enhances their resistance to antimicrobial and biocidal agents making infections difficult to treat. Mechanisms of biofilm-induced antimicrobial resistance involve reduced penetration of antimicrobial agents, increased expression of efflux pumps, altered microbial physiology, and genetic changes in the bacterial population. Factors contributing to the formation of biofilms include nutrient availability, temperature, pH, surface properties, and microbial interactions. Biofilm-associated infections can have serious consequences for patient outcomes, and standard antimicrobial therapies are often ineffective against biofilm-associated bacteria, making diagnosis and treatment challenging. Novel strategies, including antibiotics combination therapies (such as daptomycin and vancomycin, colistin and azithromycin), biofilm-targeted agents (such as small molecules (LP3134, LP3145, LP4010, LP1062) target c-di-GMP), and immunomodulatory therapies (such as the anti-PcrV IgY antibodies which target Type IIIsecretion system), are being developed to combat biofilm-induced antimicrobial resistance. A multifaceted approach to diagnosis, treatment, and prevention is necessary to address this emerging problem in healthcare settings.

## 1 Introduction

**Figure 1:**
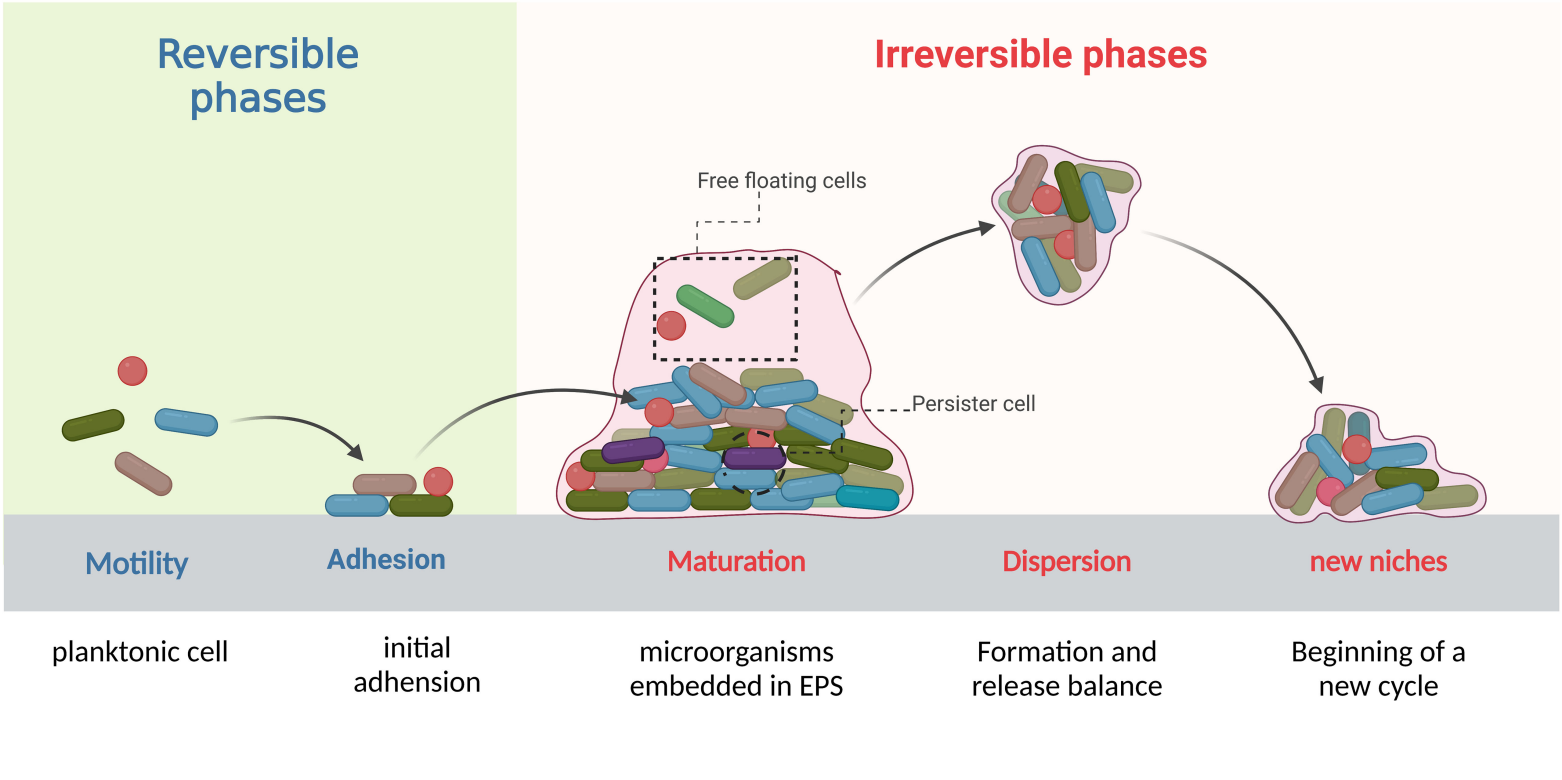
Life cycle of biofilm: Expanding the conceptual model of biofilm formation.

Biofilms are complex structures formed by communities of microorganisms that attach to biotic and/or abiotic surfaces. With the continuous influx of new colonizers and outflow of old colonizers, the formation and dispersion occur in a periodical cycle that makes biofilm an open system (Ma et al., 2022; Sauer et al., 2022). This development cycle contains complex and progressive processes which are divided into four stages: initial adhesion, formation of microcolonies, biofilm maturation, and detachment and dispersion (**Figure 1**). The process of biofilm formation is reversible in the initial stages and depends on environmental conditions but once the colonies pass the initial attachment and adhesion phase, it can lead to irreversible attachment to different surfaces (Caixeta Magalhães Tibúrcio et al., 2022; Sauer et al., 2022). Later, genetic and phenotypic changes take place within the bacteria encapsulated in the matrix of biofilm and lead to biofilm-induced resistance. We divide these changes into physical mechanisms and biological mechanisms. Physically, bacteria produce a thick biofilm matrix to evade antimicrobial agents (Flemming et al., 2023). Biological mechanisms involve the upregulation of efflux pumps (Lewis et al., 2023), cyclic di-GMP regulation (Yu et al., 2023), quorum sensing system (Oliveira et al., 2023), presence of persister cells (Kouhsari et al., 2023), and horizontal gene transfer (Moura de Sousa et al., 2023). These changes occur in bacteria potentially increasing their ability to survive from antibiotics and other life-threatening conditions, and might also enable them to be multi-drug resistance strains. Unfortunately, biofilms have been identified in various clinical settings (Chakraborty et al., 2021; Jakubovics et al., 2021) and pose significant challenges for biofilm-mediated infection management, including difficulties in diagnosing and treating infections caused by biofilm forming bacteria, increased risk of persistent and recurrent infections, and the potential for the spread of resistant bacteria to other patients and healthcare settings. The objective of this review summarizes mechanisms of biofilm-induced resistance, bacteria involved in biofilm formation and biofilm-related diseases, and provides novel precise target strategies which can be effective in the treatment of biofilms-induced infections.

Recently bacterial biofilm has been proven to be an aggressive strategy employed by bacteria, which can not only provide shelter for bacteria in extreme environments like the immune system but also able to attack them (Bordon, 2023).

According to the study conducted by Lucia Vidakovic et al., the human pathogen *Vibrio cholerae* forms biofilms on the surface of various human immune cells, which encases the immune cells and kill them with a high local concentration of a secreted hemolysin before the biofilms disperse in a c-di-GMP-dependent manner (Vidakovic et al., 2023). Besides, differences in biofilm matrix composition and the mechanism of surface attachment are found between biofilms on macrophages and other surfaces. Although many bacterial pathogens can form biofilms during infections, knowledge of whether other pathogens can have similarities in biofilm formation and extra function on immune cells remains unknown. While multispecies biofilms often have distinct properties from single species biofilms, little is known about modulation and interactions among such commensals. A study by Liju Wang et al. shows the interspecies biofilm matrix between *Staphylococcus aureus* and streptococcus is able to mediate polymicrobial interactions (Wang et al., 2023). Another research by Wucher et al. examined how interactions among bacteria at the start of biofilm growth influence the eventual multispecies biofilm architecture and thereby determine predator access to cells within the community (Wucher et al., 2023). Now is the time for us to understand the complex interactions between microorganisms and their host, which is essential in identifying new therapeutic targets for the prevention and treatment of chronic infections. This review summarizes biofilm formation in biofilm-induced antimicrobial resistance in healthcare and provides possible ways to prevent biofilm formation or eliminate mature biofilms.

## 2 Mechanisms of antimicrobial resistance in biofilms

Bacteria within a biofilm are embedded in a matrix of extracellular polymeric substances (EPSs), now may also be presented as “matrixome”, which primarily consists of polysaccharides, proteins, water-insoluble compounds, and lipids. Interactions among EPS molecules and the bacteria surrounded by them are complex, but closely related to antimicrobial resistance in biofilms that involves both physical and biological mechanisms (Flemming et al., 2023).

### 2.1 Physical mechanisms of antimicrobial resistance in biofilms

#### 2.1.1 EPSs and environmental factors

EPSs are dynamic in space and time and the inclusive components interact in involute ways. This enables EPSs to fulfill various functions: (i) stabilize the matrix (ii) acquire nutrients (iii) modulate chemical gradients and (iv) maintain microenvironments within biofilms. As a physical barrier of a biofilm, EPSs prevent antimicrobial agents from reaching the bacterial cells by physically hinder the penetration of antimicrobial agents and decrease their effectiveness. The effects of EPSs on the diffusion of antibiotics have different outcomes based on drug concentration: (i) subinhibitory level of antibiotics may not trigger the defense of bacteria but can be hindered by the formation of EPS (ii) slow growth rate within biofilm weaken the interaction between antibiotics and bacteria, and antibiotics can be removed by efflux pumps,(iii) the antibiotic effect weakens the bacteria that cause absorption, which seems to be a defensive effect of the bacteria, and (iv) slowing down of diffusion decreases (Kosztołowicz and Metzler, 2020). The low penetration rate can lead to inadequate concentrations of antimicrobial agents within the biofilm, and reduce their efficacy. Till now, the information on EPSs is mostly gained from single-species biofilms grown under controlled laboratory conditions. Although we are now short of knowledge in the context of polymicrobial biofilms, the formation, maintenance and transformation of EPSs are identified to be highly regulated by genetic control (cyclic-di-GMP, quorum sensing molecules, small RNAs)and environmental factors (such as oxygen, temperature, nutrients, osmolarity, etc.) (Flemming et al., 2023). Genetic factors we will discuss in the later part of the article.

Environmental factors that affect biofilm formation, include hypoxia, which can create an infectious hypoxic environment that promotes virulence gene expression (André et al., 2022); acidosis can alter the composition of biofilms, with low pH levels that can impair immune system function (Dalwadi and Pearce, 2021; Behbahani et al., 2022). Besides, iron metabolism plays a crucial role in biofilm formation and antibiotic resistance in various pathogenic microorganisms (Zhang et al., 2021; Liu K et al., 2022). A recent study found that iron availability significantly enhances the formation of biofilm structures, suggesting that targeting iron metabolism may disrupt biofilm formation and increase the effectiveness of antibiotic treatment (Liu K et al., 2022).

But different species manage their regulation in different ways, and there is no mutual regulatory pathway for all EPS components in all biofilms of various bacterial strains. Since the research on mixed species biofilms is limited, it is imagined that managing biofilms in technical systems intending to upper-or lower-produce EPS is not feasible. In addition, it is still challenging to identify which member in a given biofilm community produces certain components at what time point and for what function. The existent methods of analysis have merely revealed snapshots of the matrix only. However, what triggers or inhibits the production of specific EPS molecules, and how it interacts with different community members remains unexplored. Besides, bacterial biofilms may display changes in bacterial cell envelop due to the production of a slimy outer layer or the formation of thicker cell walls (Vandana and Das, 2022). These modifications can reduce the permeability of the cell envelope to antimicrobial agents, making it more difficult for them to penetrate the cells.

### 2.2 Biological mechanisms of antimicrobial resistance in biofilms

#### 2.2.1 Efflux pumps

**Figure 2:**
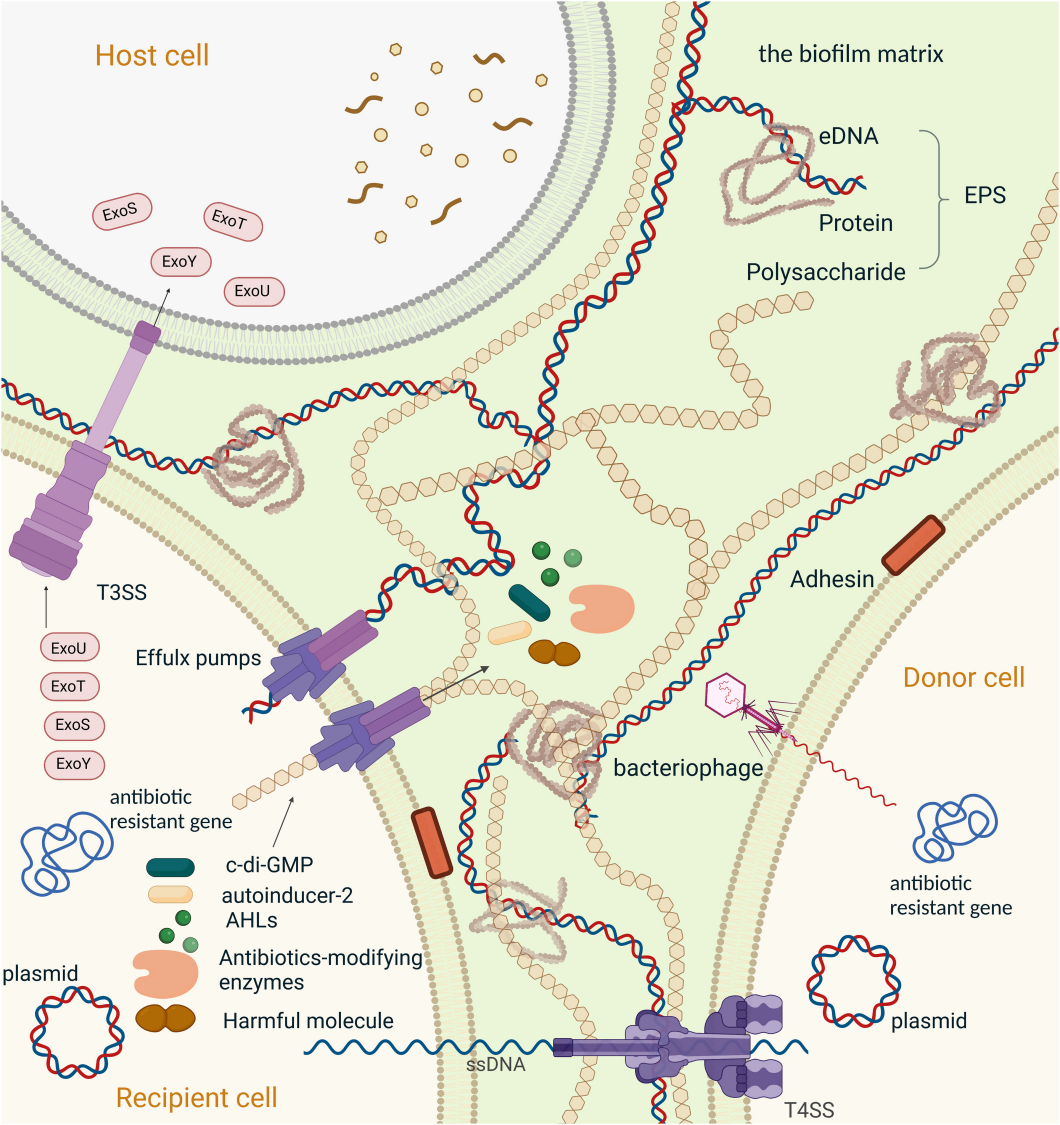
Mechanisms of resistance to antimicrobial agents in a biofilm,Created with BioRender.com. Schematic overview of the major mechanisms involved in antimicrobial resistance employed by bacterial biofilms. (1) The second messenger molecule, c-di-GMP, is associated with biofilm formation, virulence, and metabolic activity; Individual bacteria can synchronize their gene expression and alter their behaviour through chemical interactions between organisms in microbial communities, which is generally referred to as quorum sensing (QS)system. (2)N-Acyl homoserine lactones (AHLs) mediated QS system, the main QS systemin Gram-negative bacteria;(3) Autoinducer-2(AI-2) is the most common QS signal that mediated communication between intraspecies and interspecies; (4) Antibiotics-modifying enzymes induce resistance to multiple antibiotics, specially β-lactams, by transforming antimicrobials to a non-toxic form. (5) Efflux pump plays an important role in antibiotic resistance, including the efflux of the functional molecules (c-di-GMP, AHLs, AI-2 and antibiotics-modifying enzymes). extracellular polymeric substances (EPS), also efflux harmful ingredients (such as antibiotics and the metabolic intermediates) out of the bacteria. And is proven to be involved in the regulation of aggregation of the microcolonies and gene expression within biofilm; Horizontal gene transfer (HGT) is the process that passing antibiotic-resistant genes between bacterium. Here we present HGT in two ways: (6) conjugation between bacterium transmitted by plasmids and T4SS, (7) transduction between bacterium transmitted by bacteriophages. (8)T3SS in associated with phagocytic avoidance, cytotoxicity and systemic spread of bacteria. And is function by injecting effectors into the host cell directly, here we take effectors of Pseudomonas aeruginosa (ExoS, ExoT, ExoU, ExoY) as an example. (9) The components of EPS, including extracellular DNA, extracellular proteins and polysaccharides, hinder drug penetration and therefore cause drug resistance in biofilm.

Biofilm-associated resistance can be attributed to the upregulation of efflux pumps such as *AcrAB-TolC* in *E. coli*, *MexAB-OprM* in *P. aeruginosa*, *AdeFGH* in *A. baumannii*, and *AcrD* in *S. enterica*. They play a key role in the develop of drug resistance in bacteria:(i) efflux drugs to reduce the concentration, (ii) efflux molecules from EPSs to facilitate biofilm matrix formation and (iii) efflux quorum sensing (QS) molecules to regulate QS system. QS is a system that bacteria utilize to synchronize their gene expression and form a biofilm, which we will explain in the later part. Efflux pumps indirectly regulate genes involved in biofilm formation, antibiotic molecules and metabolic intermediates, and influence aggregation by promoting or preventing adhesion to surfaces and other cells (Stephen et al., 2022). Bacteria in the biofilm can upregulate the expression of efflux pumps in response to antimicrobial exposure, thereby reducing the intracellular concentration of the drug (**Figure 2**). Efflux pumps often have a broad spectrum of activity and can extrude multiple classes of antimicrobial agents, making them important targets to control antibiotic resistance.

To date, eight classes of efflux pump inhibitors (EPIs) have been reported (**Table 1**). (i) One major class of EPIs display broadspectrum efflux pump inhibitory activity by competitive inhibition (such as Phenylalanine-Arginine-β-Naphthylamide (PaβN)) (Salehi et al., 2021). (ii)Another class of EPIs act directly on the AcrAB-TolC efflux pump by binding to the pocket in the TM region of the L promotor of AcrB (like Pyridylpiperazine-based allosteric inhibitors) (Plé et al., 2022). (iii)EPIs may also function by binding to the “hydrophobic trap” in the T promotor of asymmetric AcrB. This type of inhibitor includes three classes of EPIs, pyridopyrimidine (such as D13-9001 (Kumar Roy and Patra, 2020), the pyranopyridine derivatives (like MBX series (Vargiu et al., 2014), and benzo[h]chromene compounds (Wang et al., 2021). (iv)Also, EPIs can deactivate efflux pumps via binding to site III of AcrA within the AcrAB-TolC complex,like NSC-33353 (D’Cunha et al., 2021). (v)And EPI TXA compounds (such as TXA09155) act by membrane disruption and support efflux inhibition (Zhang et al., 2022b). (vi)Finally, Arylpiperazines can increase the intracellular accumulation of antibiotics (Casalone et al., 2020). Each of the EPIs above can boost the efficiency of one or more antibiotics, but the effectiveness of the eight classes of EPIs has not been compared in parallel groups. However, a recent study suggests that the role of the RND efflux pumps *AdeABC*, *AdeFGH*, and *AdeIJK* in biofilm formation may not be crucial, indicating that these pumps may not be promising targets for biofilm inhibition (Abd El-Rahman et al., 2023).

**Table 1:** Mechanisms of biofilm-induced antibiotic resistance and inhibitors.

Mechanism	Effects	Inhibitors	Inhibitory activity	Target bacteria
c-di-GMP	i)High level of c-di-GMP is associated with biofilm formation, low level of c-di-GMP is associated with a planktonic lifestyle. ii)High level of c-di-GMP forces bacteria to rapidly produce biofilm matrix products with high energy consumption, as a result, bacteria in the biofilm enter a subsequent low metabolic state.	small molecules(LP3134, LP3145,LP4010,LP1062) (Andersen et al., 2021a; Dong et al., 2022; Lichtenberg et al., 2022)	Antagonize DGC enzymes that synthesise c-di-GMP	*Pseudomonas aeruginosa*
		ebselen, ebselen oxide(Lieberman et al., 2014)	Ebselen and ebselen oxide are both inhibitors of c-di-GMP binding to receptors containing an RXXD domain including PelD and diguanylate cyclases (DGC). They both function by covalently modifying cysteine residues.	*Pseudomonas aeruginosa*
T3SS	Inject effectors (ExoS, ExoT, ExoU, ExoY) into host cells	V2L2MD (immunotherapeutic antibodies)(Warrener et al., 2014)	Target PcrV tip protein	*Pseudomonas aeruginosa*
		natural compounds (12(6.4),12(4,6)) (Ngo et al., 2019)	Prevent the binding of PscF to PscE-PscG (two cognate chaperones of the T3SS needle subunit protein PscF)	*Galleria mellonella*
LuxI/R gene (AI-1 QS system)	Primarily mediated by AHL, controls quorum-sensing-controlled gene expression in a tandem regulatory way.	Quercetin (Gopu et al., 2015; Chittasupho et al., 2021)	Act as a competitive inhibitor for signalling compounds in LasR receptor pathway.	*Pseudomonas aeruginosa, Klebsiella pneumoniae*
		azithromcin/PAβN(Phenylalanine-Arginine-β-Naphtylamide,1)(Elsheredy et al., 2021)	inhibit the production of autoinducer C4HSL and four QS-dependent virulence factors (*lasI, lasR, Rh1I and rhiR)*	*Pseudomonas aeruginosa*
Lus/AI-2 QS system	mediate interspecies quorum sensing and intraspecies communication by AI-2	5-Fluorourcil (Sedlmayer et al., 2021)	A quorum-quencher	*Staphylococcus epidermidis, Escherichia coli, Vibrio Harveyi*
Hinder drug penetration	reduce bacterial susceptibility to antibiotics	Nanoparticles combined with laser(Teirlinck et al., 2018)	Increase drug diffusion and lead to biofilm disruption by forming laser-indued vapour nanobubbles (VNBs)	Gram-positive and Gram-negative bacteria
Efflux pumps	i)efflux extracellular polymeric substances (EPSs) to facilitate biofilm matrix formation; ii) efflux quorum sensing (QS)molecules to regulate QS. iii)efflux harmful molecules; iiii) indirectly regulate genes involved in biofilm formation; iiiii) influence aggregation of bacteria	PAβN(Phenylalanine-Arginine-β-Naphtylamide,1)(Lomovskaya et al., 2001; Kaynak Onurdağ et al., 2021)	i)a competitive inhibitor of the three RND pumps of *P. aeruginosa* (MexAB-OprM, MexCD-OprJ, and MexEF-OprN). PAβN is able to potentiate the activity of different families of antibiotics, including fluoroquinolones, macrolides, oxazolidinones, chloramphenicol and rifampin, except aminoglycosides ii) PAβN inhibits the expression of EF system genes (*adeA, adeB, adeR, adeS, adeF, adeG, adeH, adeL*) in *Acinetobacter baumannii*	*Pseudomonas aeruginosa, Acinetobacter baumannii*
		Sodium Malonate (Cauilan and Ruiz, 2022)	An inhibitor of AcrAB-TolC functions by binding to multiple AcrBlocations, including the AcrB proximal binding pocket.	*Escherichia coli*
		D13-9001(Nakashima et al., 2013; Kumar Roy and Patra, 2020)	A specific inhibitor of MexAB-OprM of *P. aeruginosa* via binding to MexB and AcrB in the deep binding pocket region known as the hydrophobic trap	*pseudomonas aeruginosa*
		MBX2319(Vargiu et al., 2014)	MBX2319 binds to the lower part of the distal pocket in the B protomer of AcrB, impairing the proper binding of substrates	*Enterobacteriaceae*
		2H-benzo[h]chromene(Wang et al., 2021; Guo et al., 2023)	C-2 position of 2H-benzo[h]chromene binds to the hydrophobic trap to impair the function of the AcrB-mediated efflux pump	*Escherichia coli*

#### 2.2.2 Second messengers

Cyclic di-GMP (c-di-GMP) is a second messenger molecule that has an impact on the biofilm formation and metabolism of bacteria. When the concentration of c-diGMP is high, it promotes biofilm formation, while low levels lead to a planktonic lifestyle, and also influence the antimicrobial resistance of the bacteria (**Table 2**). High c-di-GMP level in biofilm cause rapid energy spent on the production of the biofilm matrix components, and results in a subsequent low metabolic state of bacteria in mature biofilms, to some extent, which is a c-di-GMP regulated survival strategy opted by most bacteria (Feng et al., 2020; Lichtenberg et al., 2022).

**Table 2:** Infections associated with biofilm .

Biofilm infections	Microscopic changes and paraclinical changes	Common pathogens
dental caries and periodontal disease(Mosaddad et al., 2019; Valm, 2019; Moussa et al., 2022)	Microbial community transit from states of health to states of dysbiosis. Which can also be correlated with immune-mediated inflammatory disease in the distant part of the body.	*Streptococcus mutans*, *Porphyromonas* *gingivalis, Prevotella* and its species
Chronic rhinosinusitis(Wagner Mackenzie et al., 2017)	Biofilm formation is associated with ciliary destruction and mucus stasis. Persistence of infection and immune provocation may result from superantigens and direct activation of TLR-2 receptors.	*Staphylococcus*, *Propionibacterium, Corynebacterium, streptococcus*
Pseudomonas aeruginosa in Cystic fibrosis (CF)/COPD (Cantin et al., 2015; Phuengmaung et al., 2022)	Mutation of CFTR gene causes abnormality of chloride channels in mucus and sweating cells which lead to mucosal hyper concentration on the airway surfaces of CF patients. These thick mucus layer hinders the clearance of pathogenic microorganisms and. make the lung of CF patients favorable for biofilm formations	*Pseudomonas aeruginosa, Candida albicans, A. fumigatus*
chronic wounds (Khalid et al., 2023)	Biofilm formation impairs healing in chronic wounds. The spatial distribution of microbials in multi-species biofilms tougher the diagnosis and treatment of wounds.	*Staphylococci aureus, Pseudomonas* *aeruginosa, E. coli, E. faecalis, C. freundill*
Inflammatory bowel disease(IBD)(Santana et al., 2022)	A group of disorders featured with prolonged inflammation in colon and small intestine, which have been observed with endoscopic mucosal biofilm formation but possess disrupted bile acid metabolism and bacterial dysbiosis.	*enteropathogenic E. coli, Faecalibacterium prausnitzii, Bilophila*
Urinary tracts infections (UTIs) (Zhao et al., 2020)	Biofilms were formed inside the bladder epithelial cells; these intracellular bacterial communities are protected from neutrophil attack and could proliferation. Which properly contribute to chronic and reiterative UTIs.	*uropathogenic E. coli, Staphylococci aureus, Klebsiella, Enterobacter, Proteus mirabilis*
Prosthetic joint infections(Li et al., 2018)	Bacteria colonize the surface of artificial joint by forming biofilm	*Staphylococcus aureus, Staphylococcus epidermidis*

In *P. aeruginosa*, there are 41 genes responsible for the synthesis and degradation of c-di-GMP, including *PA1120*, which synthesizes c-di-GMP, and *PA2133*, which degrades it (Andersen et al., 2021b). Another important gene involved in this process is *PA5487*, also known as *dgcH*. Which is a diguanylate cyclase that helps to maintain the basal level of c-di-GMP in bacteria (Wei et al., 2019). Interestingly, *dgcH* has been found to boost the fitness of bacteria in the presence of imipenem, a commonly used antibiotic, thus contributing to the development of antimicrobial resistance in *P. aeruginosa* (**Figure 2**).

Cross-regulating between cAMP and c-di-GMP has been well studied. (Hengge, 2021). For example, cAMP has been reported to regulate the transcription of certain genes that encode c-di-GMP receptors or DGCs/PDEs. Liu et al. described a regulatory pattern in which cAMP and c-di-GMP interact to synergistically regulate biofilm maintenance through their effectors. (Liu C. et al., 2022).

#### 2.2.3 cAMP-Vfr system and T3SS

The Cyclic AMP (cAMP)-Vfr system (CVS) is a global regulator of virulence gene expression in *P. aeruginosa*, including the type III secretion system (T3SS), which is a complex molecular syringe that injects effectors into host cells. The T3SS is a crucial virulence determinant and is composed of three main parts: the basal body, the needle, and the translocon (Ngo et al., 2019). Four effectors have been identified, including ExoS, ExoT, ExoU, and ExoY, which manipulate host cell signaling and/or cause cytotoxicity (Khodayary et al., 2019).

T3SS genes are organized into 10 transcriptional units, each of which is controlled by an *ExsA*-dependent promoter. Vfr, a cAMP-dependent DNA-binding protein, plays a significant role in regulating virulence gene expression, specifically type IV pili and T3SS (Janssen et al., 2020). *ExsA*, a member of the *AraC/XylS* family of transcriptional regulators, serves as the central regulator of T3SS gene expression (Janssen et al., 2020). In the latest study, citrus peel extract from Jeju Island (CPEJ) can inhibit bacterial biofilm formation (Kim et al., 2023). The result shows that CPEJ can significantly reduce the c-di-GMP level through increased phosphodiesterase activity, which suggests the potential of CPEJ for applications in clinical.

#### 2.2.4 Quorum sensing

Quorum sensing (QS) is a process by which bacteria can communicate with each other and coordinate their behavior. It can influence the formation and maintenance of biofilms, as well as the expression of genes involved in antimicrobial resistance. For example, some biofilm bacteria can produce QS-dependent enzymes that modify or degrade antimicrobial agents, reducing their effectiveness. The QS system is divided into four types, each using different signal molecules, including N-Acyl homoserine lactones (AHLs), such as autoinducer-2 (AI-2), and diffusible signaling factor (DSF) (Huang et al., 2022).

The LuxI/R signaling pathway mediates quorum sensing using N-Acyl homoserine lactones (AHLs), which tandemly regulate quorum-sensing-controlled gene expression (Yu et al., 2019). In *P. aeruginosa*, *LasI* produces and *LasR* responds to the autoinducer 3OC12–HSL. The LasR:3OC12–HSL complex activates the transcription of many genes including *RhlR*, which encodes a second quorum-sensing receptor. *RhlR* binds to the autoinducer C4–HSL, the product of *RhlI*. RhlR: C4HSL also directs a large regulon of genes, some of which are also members of the *LasR* regulon. This tandem regulatory arrangement allows LasI/R to control the first wave of quorum-sensing–controlled gene expression and RhlI/R to control the second (Yu et al., 2019). The LuxS/AI-2 QS system uses AI-2 to mediate interspecies quorum sensing and controls virulence and biofilm formation in various human pathogens (Sedlmayer et al., 2021). Mutations in different QS systems can impact bacterial fitness and virulence (Jayakumar et al., 2022).

Maryam Alshammari et al. have employed CRISPR/Cas9-HDR system to target quorum sensing and adhesion genes in *Escherichia coli* (Alshammari et al., 2023). The result indicates that the knockout of *luxS*, *fimH*, and *bolA* genes reduced EPS matrix production, which is considered the main factor in adherence, cell aggregation, and biofilm formation.

#### 2.2.5 Persister cells

Another challenge associated with biofilm infections is the presence of persister cells. Persisters are a subpopulation of antibiotic-tolerant bacterial cells that are often slow-growing or growth-arrested (Fisher et al., 2017), but this slow growth rate is reversible under lethal stress. The presence of persister cells contributes to the recalcitrance and relapse of persistent bacterial infections. A recent research has confirmed the ability of persister cells in *Pseudomonas aeruginosa* to evade the innate host response and to contribute to chronic infection (Hastings et al., 2023). Also, it is related to an increase in the risk of antimicrobial resistance in biofilm associated infections.

These phenotypes can occur through several mechanisms, including stress response (RpoS-mediated), toxin-antitoxin (TA) systems, inhibition of ATP production, reactive oxygen species (ROS), efflux pumps, bacterial SOS response, cell-to-cell communication and stringent response (ppGpp- mediated) (Kouhsari et al., 2023). Once formed, persisters can survive exposure to high concentrations of antimicrobial agents, and can remain viable for extended periods of time, even in the absence of nutrients or growth factors (Soares et al., 2019). Fortunately, there is a possible way to wake up these “sleeping bombs” to back to growth. Another study found that reactive nitrogen species (RNS) is produced by the host in response to Salmonella infection after persister formation in macrophages, which intoxicate the TCA cycle of persisters and struck them in the growth arrest stage through lowering cellular respiration and ATP production (Ronneau et al., 2023). This research indicates that inhibition of RNS production can force persister cells to regrow during antibiotic treatment which will boost the effects of antibiotics and facilitate their eradication.

#### 2.2.6 Horizontal gene transfer

Recent studies have highlighted the role of horizontal gene transfer in biofilm-associated antimicrobial resistance (Michaelis and Grohmann, 2023). Horizontal gene transfer can facilitate the spread of resistance genes among bacterial populations within biofilms, leading to the emergence of multidrug resistant strains. Mobile genetic elements, such as plasmids, transposons, and integrons, can transfer antimicrobial resistance genes between bacterial species and strains (**Figure 2**). A better understanding of the mechanisms of antimicrobial resistance in biofilms is necessary to develop more effective strategies for the prevention and treatment of biofilm-associated infections. New approaches that target the physical and biological barriers to antimicrobial penetration, as well as the mechanisms that contribute to the formation and maintenance of biofilms, may be required to overcome the challenges posed by biofilm-associated antimicrobial resistance. Additionally, the development of new antimicrobial agents or the repurposing of existing drugs may also be necessary to combat biofilm infections, particularly those caused by multidrug-resistant strains.

## 3 Biofilm-associated infections

Biofilm-associated infections are a significant healthcare challenge, as they are often resistant to conventional antibiotics and can lead to chronic infections. They include different conditions such as oral infection, infection in upper and lower airways, wounds, gastrointestinal infections, urinary tract infections (UTIs) and prosthetic joint infections (PJIs).

### 3.1 Oral infection

The oral cavity contains various microbial communities living as biofilms, which can lead to dental caries and periodontal disease (Valm, 2019) (**Table 1**). The transition from health to dysbiosis is caused by changes in community structure, both *in vivo* and *in vitro*(O’Connell et al., 2020; Moussa et al., 2022).

### 3.2 Infection in airways

There is strong evidence suggests that bacterial biofilm attached to sinonasal mucosa has played a potential role in the pathogenesis of chronic rhinosinusitis (CRS), an infection in the upper airways (Vickery et al., 2019), where *Staphylococcus aureus* has been implied to be the major pathogen. Besides this, fungal-bacterial interaction has been observed to have affected the virulence of fungi and bacteria and host immune responses (Shin et al., 2023). Although CRS manifests in self in a variety of pathogenic patterns, there is a piece of increasing evidence suggesting that it is associated with sinonasal dysbiosis and the presence of biofilm (Sabino et al., 2021).

As to the lower respiratory tract infections, repeated episodes of infection were caused by *Pseudomonas aeruginosa* in Cystic fibrosis (CF). Which is an autosomal recessive disease characterized by abnormality of chloride channels due to mutations in Cystic Fibrosis Transmembrane Conductance Regulator (CFTR) gene. Recently, rapid synergistic biofilm formation of *Pseudomonas aeruginosa* and *Candida albicans* has been observed *in vitro* and mice (Phuengmaung et al., 2020; Phuengmaung et al., 2022). These biofilms were more prominent than those induced by *Pseudomonas aeruginosa* alone, which supports *Candida*-enhanced *Pseudomonas* growth. Besides, mixedspecies biofilm was attenuated by N-acetyl-1-cysteine (Phuengmaung et al., 2020).

### 3.3 Wounds

In chronic wound infections, biofilms consisting of *S. aureus* and *P. aeruginosa* are commonly found together leading to delayed healing and persistent infections (Borges et al., 2022). Laboratory models developed to monitor biofilm colonization in chronic wounds and biopsies reveal that bacteria form species specific aggregates in distinct regions of the tissue (Khalid et al., 2023). Real-time detection of volatile metabolites can be used to discriminate between different microbial species in biofilms (Slade et al., 2022).

### 3.4 Gastrointestinal infections

Biofilm formation is a crucial factor in the development of inflammatory bowel disease and urinary tract infections. The presence of mucosal biofilms disrupted bile acid metabolism and bacterial dysbiosis in the gut microbiome of Inflammatory bowel disease (IBD) patients (Baumgartner et al., 2021). Previous result link the presence of biofilms to a dysbiotic gut microbiome, including the overgrowth of Escherichia coli and Ruminococcus gnavus (Baumgartner et al., 2021).

### 3.5 Urinary tract infections

In UTIs, biofilm formation is a major mechanism used by bacteria and is significantly correlated with virulence factors and antibiotic resistance (Zhao et al., 2020). Multidrug-resistant uropathogenic bacteria have an increasing impact on UTI management (Mark et al., 2021). Programmable probiotics show promise as a therapeutic method for treating gut-related diseases (Zhou et al., 2022).

### 3.6 Prosthetic joint infections

PJIs occur when bacteria colonize the surface of an artificial joint, such as a hip or knee replacement, and form a biofilm that makes them more resistant to antibiotics and the body’s immune response. PJIs can occur through several routes, including contamination during surgery, hematogenous seeding from another site of infection, or direct extension from adjacent infected tissue (Ahmed et al., 2019). Biofilm formation on prosthetic joints reduces the effectiveness of treatment of PJIs that lead to significant morbidity and mortality. For further therapeutic benefit, new strategies to prevent, diagnose, and treat biofilm-associated infections are needed to be explored.

### 3.7 Microorganisms involved in biofilm-related infections

#### 3.7.1 Pseudomonas aeruginosa

*P. aeruginosa* displays resistance to multiple antibiotics, including aminoglycosides, quinolones and β-lactams (Jurado-Martín et al., 2021). Biofilm formation is essential for its tremendous ability to adapt to altered environments. The main virulence factors *of P. aeruginosa* consist of: biofilm formation ability, three types of QS(Las, RhI and Pqs), efflux pumps, flagella, type IV pili, T3SS, type VI secretion system, and type II secretion system (Jurado-Martín et al., 2021).

#### 3.7.2 Acinetobacter baumanni

*A. baumannii* can form biofilm on medical surfaces, such as catheters, endotracheal tubes, and ventilators, enables its persistence in hospitals (Roy et al., 2022). The clinical outcome of patients with Carbapenem-resistant *A. baumannii* and *K. pneumoniae* is associated with high mortality rates, particularly in the mid-south region of China, and predominantly belongs to ST457 *A. baumannii* (Li et al., 2020). In addition to biofilm formation, *A. baumannii* acquires antibiotic-resistant genes via horizontal gene transfer (HGT), allowing it to evade the immune system and gain an advantage in hospitals. Antibiotic-resistant genes are transferred via conjugation, transformation, bacteria phage-mediated, nanotube-mediated, or outer membrane vesicles (Roy et al., 2022). *A. baumannii* strong biofilm-forming ability, high efficiency of HGT, and the expression of efflux pumps and antibiotic modifying enzymes render the pathogen resistant to multiple drugs, including carbapenems, aminoglycosides, and fluoroquinolones.

#### 3.7.3 Klebsiella pneumoniae

KP is a Gram-negative facultative anaerobic pathogen with striking virulence factors consisting of adhesive fimbriae, capsule, lipopolysaccharide (LPS), and siderophores or iron carriers. The carbapenem-resistant *Klebsiella pneumoniae* (CRKP) has enzymes such as *bla*_KPC-2_ which is one of the most observed carbapenemase genotypes, followed by *bla*_NDM_, *bla*_OXA-48-like,_ and *bla*_IMP_ (Wang et al., 2022). The dominant carriage pattern of the antibiotic resistance genotype is the carriage of a single carbapenemase gene. However, the combination of a single genotype results in hypervirulence in bacteria and the convergence of hypervirulence and carbapenem resistance (Roy et al., 2022).

#### 3.7.4 Escherichia coli

*E. coli* has been observed to possess many virulence factors, especially uropathogenic *E. coli* (UPEC), which include: immune suppressors (invasins like the *sisA* and *sisB*), adhesins (*fimH* adhesion, P-fimbrial adhesins) (Bunduki et al., 2021). Tetracycline resistance in *E.coli* is generally very high, followed by quinolones and β-lactams. The results show that *shiA* is the gene mostly associated with virulence, followed by *CSH, fimH/MSHA, traT, sisA, iucD, iutA, kpsMTI and PAI* (Bunduki et al., 2021).

#### 3.7.5 Enterococcus faecium

In the case of *Enterococcus faecium* the *efa* gene was the most frequently observed virulence gene, followed by *ace, esp, ebp, cylA, hyl, asa1, gelE, sprE, fsrC, fsrA and fsrB* (Gök et al., 2020). And the highest resistance rate has been observed in ciprofloxacin. Also, *Enterococcus faecium* is highly resistant to ampicillin, streptomycin, gentamicin, vancomycin, teicoplanin and linezolid (Gök et al., 2020).

#### 3.7.6 Staphylococcus aureus

In the case of Gram-positive bacteria, *Staphylococcus aureus* is the most commonly isolated pathogen in biofilm-related infections and the leading cause of numerous diseases (such as bacteremia, infectious endocarditis, infections of the skin, soft tissue, pleuropulmonary and the periprosthetic joint infection) (Pietrocola et al., 2022). Which is also the major colonizer of a medical device. An identified mutation in the genome is associated with enhanced biofilm production in *S. aureus in vitro*, that high-level expression of *manA* and *fruB* corrected biofilm deficiencies and enhanced biofilm formation (Long et al., 2023). The emergence of methicillin-resistant *Staphylococcus aureus* (MRSA) strains, which is the result of the acquisition of *mecA* gene coding for penicillin-binding protein-2a that blocks inhibitory action on peptidoglycan cross-linking, has prompted research into potential anti-virulencetargeted approaches. The strong pathogenicity of *S. aureus* due to its large repertoire of toxins (such as α-hemolysin, exfoliative toxins, and Panton-valentine leucocidin) (Ahmad-Mansour et al., 2021), also results from its facultative anaerobe characteristics (Vudhya Gowrisankar et al., 2021). As one of the main efflux pumps, NorA is an efficient multidrug-resistant system that could enhance drug resistance by increasing drug efflux, thus appearing to be an important target for efflux pump inhibitors such as quino-4-carboxamide derivatives (3a and 3b), which enhanced the bactericidal activities of ciprofloxacin and fluoroquinolone via NorA inhibition *in vitro* (Cannalire et al., 2020). There are also plenty of novel inhibitors that have been proven effective *in vitro* (Cernicchi et al., 2021), but they widen the gap between clinical application and experimental achievement.

## 4 Discussion

### 4.1 Detection and monitor of biofilm formation

Recent research has shown that biofilm growth is a developmental process resembling developmental processes in multicellular eukaryotes (Futo et al., 2022). But studying the ontogeny of biofilm *in vivo* is still problematic since it is difficult to develop *in vitro* models of biofilm formation that mimic the internal environment. As a result, the traditional four-step model of biofilm formation is not suitable for biofilm-mediated infections, which has failed to capture many aspects of bacterial biofilm physiology, especially in clinical settings (Sauer et al., 2022). Karin Sauer et al., have presented a new developmental model for biofilm formation that may include all the diverse scenarios and microenvironments where biofilms are formed (Sauer et al., 2022). This expanded model considers biofilm as an open system with a continuous influx of new members in an external/internal environment. Although it is a work in progress based on current knowledge, this new model has interesting insight into the study of biofilm formation.

To define the dynamics and critical transitional phases of the formation process, as well as microbial interactions and colony dynamics, there is an urgent need for non-invasive methods that can provide *in vivo* visual information to record the gross morphological changes of the biofilm. Carlos Molina-Santiago et al. has detailed a novel non-invasive method for tracking bacterial growth and biofilm dynamics, Baclive (Molina-Santiago et al., 2022). However, our ability to follow complex intraspecies and interspecies interactions *in vivo* at the cellular level remained limited.

In addition, early detection and monitoring of biofilms holds significant importance for the diagnosis of biofilm-mediated infections and the prevention of further spread and new lesions. Several methods are currently used for the detection of bacterial communities in biofilm in controlled laboratory settings, including molecular methods (such as quantitative polymerase chain reaction (q-PCR)); microscopy techniques (such as confocal laser scanning microscopy (CLSM), scanning electron microscopy (SEM)); spectroscopic method (Raman spectroscopy and surface-enhanced Raman spectroscopy); identification and localization of microorganisms in biofilm (such as fluorescence *in situ* hybridization (FISH)). Various FISH techniques related to biofilm have been developed. For example, FISH can be modified to catalyzed reporter deposition-FISH (CARD-FISH), peptide nucleic acid (PNA)-FISH, locked nucleic acids (LNA)-FISH that allow multiple bacteria to be identified at the same time (Azeredo et al., 2017). However, these methods are limited in biofilm detection in clinical settings (Xu et al., 2020).

Several new approaches have been recently developed for biofilm studies which can contribute to a more comprehensive understanding of biofilm physiology, structure and composition. One promising approach is optical coherence tomography (OCT), a non-contact method for imaging the topological and internal microstructure of samples in three dimensions (Bouma et al., 2022). In a recent study, OCT has been used to differentiate pathogenic bacteria and biofilms in otitis media (Locke et al., 2022). Another study uses longitudinal catheter-based 3-D OCT to monitor biofilm formation in endotracheal tubes (ETTs) (Dsouza et al., 2021), their results indicate that catheter-based OCT provides a non-invasive way to identify biofilm within the tube and can differentiate biofilm-positive from biofilm negative groups. Moreover, the OCT image-based features obtained can offer possible biomarkers to detect biofilm *in vivo* (Dsouza et al., 2019). Biofilm associated biomarkers are unique molecules produced during biofilm growth (such as specific proteins on bacterial cells, metabolites), or stimulated by biofilm specific host responses (such as antibodies). That can be detected using standard methods (Xu et al., 2020). Elisabeth A. Slade et al. presents a real‐time detection of biofilms in a clinical wound infection model by detecting volatile metabolites for species‐level discrimination (Slade et al., 2022). The volatile compounds successfully differentiate between the pathogens studied, which can be developed as rapid point‐of‐care diagnostics for wound infection. Although studies are limited to biomarkers that are unique to bacterial biofilms, especially those present in multiple bacterial species, but detection of biofilm matrix components (such as cellulose (Perumal et al., 2021)) and QS signal profiling might discover potential biomarkers. Other novel approaches, including targeted nanopore sequencing (TNPseq) (Zhang et al., 2022a); metagenomics (Taş et al., 2021); and nanoparticles (Funari and Shen, 2022).

### 4.2 Treatment strategies

Biofilm targeting therapies can help to prevent and treat bacterial infections by inhibiting the formation of biofilms. One approach is to target the cyclic-di-GMP pathway, which involves inhibiting diguanylate cyclase (DGC) enzymes that synthesize c-di-GMP (Andersen et al., 2021a). Small molecules have been identified that can disperse *P. aeruginosa* and *A. baumannii* biofilms without toxic effects on eukaryotic cells. Another approach targets T3SS which can cause severe tissue damage and inhibit wound repair. Antibodies and natural compounds can interfere with the T3SS structure and/or its function (Ngo et al., 2019; Ranjbar et al., 2019). The cytostatic anti-cancer drug 5fluorouracil (5-FU) has been shown to inhibit AI-2 production and release by MRSA, *S. epidermidis*, *E. coli*, and *V. harveyi*, and could potentially be used as an anti-infective therapy (Sedlmayer et al., 2021). CRISPR/Cas9-HDR system may provide an efficient and site-specific gene editing approach which targets the QS mechanism and adhesion property to suppress biofilm formation (Alshammari et al., 2023).

Novel strategies for biofilm-related infection management include (i) the use of nanoparticles combined with a laser to improve drug diffusion (Teirlinck et al., 2019), (ii)pH-responsive drug delivery systems (Ding et al., 2022), and (iii)targeting the extracellular polymeric substances (EPS) in a mature biofilm to expose the bacteria and permit antibacterial agents to reach the biofilm (Han et al., 2022). Moreover, interfering with iron metabolism can also disrupt iron-dependent biological processes by binding iron-utilizing proteins (Ribeiro et al., 2022). (v) Apart from that, the dispersal of biofilm produced by multi-drug resistant bacteria can be achieved through phage therapy. (Pallavali et al., 2021).

In all, real-time detection and non-invasive biofilm detection of biofilm, as well as biofilm specific labels are to be discovered. With increased sensitivity in signal detection of biofilm formation, localized imaging is for certain to be combined with a therapeutic benefit. In the future, more experiments are required to explore more regulation of biofilm formation and dispersal, in order to induce the dispersal of mature biofilms and restore the bacteria planktonic growth. Besides, ongoing studies show that inhibition of c-di-GMP and cAMP synthesis, as well as interfering QS system can prevent the maintenance of biofilm. And destroying EPS while applying antibiotics would be an effective way to boost antibiotic efficacy. Currently, the combination of nanomaterials and antibiotics is the most feasible way to disrupt biofilms.

## 5 Conclusion

In conclusion, biofilms represent an emerging battleground in healthcare due to their ability to induce antimicrobial resistance. These microbial communities have developed complex mechanisms that protect them against the action of antibiotics and host defenses, making them challenging to eradicate. The development of biofilm-specific therapies is crucial to overcome these challenges and prevent the emergence of new antibiotic-resistant strains. Several approaches, such as the use of alternative antimicrobial agents, quorum sensing inhibitors, and biofilm disruptors, have shown promising results in laboratory studies. However, the translation of these findings into clinical practice remains a significant challenge. Further research is needed to understand the mechanisms of biofilm formation and antibiotic resistance better, identify novel targets for intervention, and develop more effective strategies for the prevention and treatment of biofilm-related infections. In summary, the battle against biofilm-induced antimicrobial resistance requires a multifaceted approach that involves collaboration between researchers, clinicians, and policymakers to mitigate the public health threat posed by these microbial communities.

## Author contributions

LP: Conceptualization, Data curation, Formal analysis, Software, Visualization, Writing – original draft. SP: Conceptualization, Formal analysis, Investigation, Software, Supervision, Validation, Visualization, Writing – original draft, Writing – review & editing. SL: Conceptualization, Resources, Writing – review & editing. FW: Funding acquisition, Project administration, Resources, Visualization, Writing – review & editing.
